# Influenza vaccine effectiveness against influenza-related hospitalization during a season with mixed outbreaks of four influenza viruses: a test-negative case-control study in adults in Canada

**DOI:** 10.1186/s12879-017-2905-8

**Published:** 2017-12-29

**Authors:** Melissa K. Andrew, Vivek Shinde, Todd Hatchette, Ardith Ambrose, Guy Boivin, William Bowie, Ayman Chit, Gael Dos Santos, May ElSherif, Karen Green, François Haguinet, Scott A. Halperin, Barbara Ibarguchi, Jennie Johnstone, Kevin Katz, Joanne M. Langley, Jason LeBlanc, Mark Loeb, Donna MacKinnon-Cameron, Anne McCarthy, Janet McElhaney, Allison McGeer, Michaela K. Nichols, Jeff Powis, David Richardson, Makeda Semret, Grant Stiver, Sylvie Trottier, Louis Valiquette, Duncan Webster, Lingyun Ye, Shelly A. McNeil, on behalf of the Public Health Agency of Canada/Canadian Institutes of Health Research Influenza Research Network (PCIRN) Serious Outcomes Surveillance Network and the Toronto Invasive Bacterial Diseases Network (TIBDN)

**Affiliations:** 1 Canadian Center for Vaccinology, IWK Health Centre and Nova Scotia Health Authority, Dalhousie University, 5850/5980 University Ave, Halifax, Nova Scotia B3K 6R8 Canada; 2 GSK, King of Prussia, Current affiliation: Novavax Vaccines, Washington, DC, USA; 3 Centre Hospitalier Universitaire de Québec, 2705 Boulevard Laurier, RC-709, Québec, Québec G1V 4G2 Canada; 4 University of British Columbia, 452D, Heather Pavilion East, VGH, 2733 Heather Street, Vancouver, British Columbia V5Z 3J5 Canada; 5 Leslie Dan Faculty of Pharmacy, University of Toronto, Current affiliation: Sanofi Pasteur, Swiftwater, Pennsylvania USA; 6 Business & Decision Life Sciences, Bruxelles, Belgium, on behalf of GSK (Wavre, Belgium), Current affiliation: GSK, Wavre, Belgium; 7 Mount Sinai Hospital, 600 University Ave, Room 210, Toronto, Ontario M5G 1X5 Canada; 8 GSK, Wavre, Belgium; 9 GSK, Mississauga, Ontario, Canada, Current affiliation: Bayer Inc, Mississauga, Ontario Canada; 10 McMaster University, Michael G. DeGroote Centre for Learning, 1200 Main Street West, Room 3208, Hamilton, Ontario L8S 4K1 Canada; 11 North York General Hospital, 4001 Leslie St, Toronto, Ontario M2K 1E1 Canada; 12 The Ottawa Hospital, Ottawa Hospital Civic Campus, 1053 Carling Ave, Ottawa, Ontario K1Y 4E9 Canada; 13 Health Sciences North Research Institute, 41 Ramsey Lake Rd, Sudbury, Ontario P3E 5J1 Canada; 14 Michael Garron Hospital, 825 Coxwell Ave, Toronto, Ontario M4C 3E7 Canada; 15 William Osler Health System, Department of Infectious Diseases and Medical Microbiology, 2100 Bovaird Dr East, Brampton, Ontario L6R 3J7 Canada; 16 McGill University, McGill University Health Centre, Glen Site, 1001 Decarie Blvd, Montreal, Quebec H4A 3J1 Canada; 17 Université de Sherbrooke, 3001 12th Ave North, Sherbrooke, Quebec J1H 5N4 Canada; 18 Saint John Regional Hospital, Dalhousie University, 400 University Ave, Saint John, New Brunswick E2L 4L2 Canada

**Keywords:** Influenza, Vaccine, Effectiveness, Adults, Hospitalization

## Abstract

**Background:**

The Serious Outcomes Surveillance (SOS) Network was established to monitor seasonal influenza complications among hospitalized Canadian adults and to assess the effectiveness of influenza vaccination against severe outcomes. Here we report age- and strain-specific vaccine effectiveness (VE) in preventing severe outcomes during a season characterized by mixed outbreaks of four different influenza strains.

**Methods:**

This prospective, multicentre, test-negative case-control study evaluated the VE of trivalent influenza vaccine (TIV) in the prevention of laboratory-confirmed influenza-hospitalization in adults aged ≥16 years (all adults) and adults aged 16–64 years (younger adults). The SOS Network identified hospitalized patients with diagnoses potentially attributable to influenza during the 2011/12 influenza season. Swabs collected at admission were tested by reverse transcriptase polymerase chain reaction (RT PCR) or viral culture to discriminate influenza cases (positive) from controls (negative). VE was calculated as 1-odds ratio (OR) of vaccination in cases versus controls × 100.

**Results:**

Overall, in all adults, the unadjusted and adjusted VEs of TIV against influenza-hospitalization were 41.8% (95% Confidence Interval [CI]: 26.0, 54.3), and 42.8% (95% CI: 23.8, 57.0), respectively. In younger adults (16–64 years), the unadjusted and adjusted VEs of TIV against influenza-hospitalization were 35.8% (95% CI: 4.5, 56.8) and 33.2% (95% CI: −6.7, 58.2), respectively. In the all adults group, adjusted VE against influenza A/H1N1 was 72.5% (95% CI: 30.5, 89.1), against A/H3N2 was 86.1% (95% CI: 40.1, 96.8), against B/Victoria was 40.5% (95% CI: −28.9, 72.6), and against B/Yamagata was 32.3% (95% CI: −8.3, 57.7). The adjusted estimate of early season VE (from November 1 to March 11) was 54.4% (95% CI: 29.7–70.4), which was higher than late season (from March 11 to May 25) VE estimate (VE: 29.7%, 95% CI: -5.3, 53.1).

**Conclusions:**

These results suggest that TIV was highly effective against A viruses and moderately effective against B viruses during a mild season characterised by co-circulation of four influenza strains in Canada. Findings underscore the need to provide VE assessment by subtype/lineage as well as the timing of vaccination (early season vs late season) to accurately evaluate vaccine performance and thus guide public health decision-making.

**Trial registration:**

ClinicalTrials.gov Identifier: NCT01517191. Registration was retrospective and the date of registration was January 17, 2012.

## Background

Numerous countries provide publicly-funded influenza vaccination programs. In addition to advocating for a particular focus on people at high risk of influenza-related complications or hospitalization, some countries, such as Australia, Canada, and United States (US) now recommend universal vaccination for those aged 6 months and older [1–3]. Despite these recommendations, the benefits of influenza vaccination remain controversial due to the variability of effectiveness of the vaccine between seasons and among individuals. Furthermore, although the seasonal influenza vaccine is expected to provide benefit, particularly against severe outcomes relating to influenza, evaluating the effect of influenza vaccination on hospitalizations and deaths in observational studies is challenging.

Hospital-based surveillance networks are used by many countries to monitor influenza disease and to assess vaccine effectiveness (VE) against severe outcomes attributable to influenza to guide public health decision-making. Prospective observational studies in adults conducted between 2010 and 2015 following the H1N1 pandemic in 2009 mostly report moderate adjusted VE against influenza-hospitalization (37–61%), although one study provided an unadjusted estimate of 33%, and another reported that influenza vaccination did not reduce the risk of influenza-related hospital admission in adults aged ≥20 years [4–10]. This variability may be due to the degree of vaccine match with the circulating strains across seasons and between regions, the virulence of the viruses circulating, unmeasured confounders, and/or differences in study design such as screening case definitions, laboratory diagnostics used, outcomes assessed, and the selection of controls.

The Serious Outcomes Surveillance (SOS) Network of the Public Health Agency of Canada/Canadian Institutes of Health Research Influenza Research Network (PCIRN) was established in 2009, at the time of the H1N1 pandemic. The objectives were to prospectively monitor serious outcomes associated with seasonal influenza, burden of influenza disease, and VE in the prevention of laboratory-confirmed influenza-hospitalization in hospitalized adults aged ≥16 years using data collection protocols designed to overcome some of the limitations of previous observational VE studies [11, 12].

In the current paper, we describe a multicentre, multi-province, test-negative case-control study of seasonal influenza VE during the 2011/12 influenza season, where Canadian national surveillance reported mixed outbreaks of four influenza strains [13]. The main objectives of this study were to evaluate the VE of trivalent influenza vaccine (TIV) in the prevention of laboratory-confirmed influenza-hospitalization both overall and by influenza strain for all adult patients (≥16 years) and for younger adults (16–64 years). Secondary objectives were to assess VE of TIV against influenza-related hospitalization (for all strains and specifically for influenza B) by month of hospital admission (early season/late season). Results from the SOS Network this season focusing specifically on influenza VE and the role of frailty in older adult patients (≥65 years) have been reported elsewhere [14].

## Methods

This prospective, multicentre, test-negative case-control study assessed the VE of seasonal TIV among hospitalized patients admitted from 1 November 2011 to 25 May 2012. This study was conducted by the PCIRN SOS Network in collaboration with the Toronto Invasive Bacterial Diseases Network (TIBDN) [11, 12]. Influenza surveillance was performed in 38 academic and community sentinel hospitals in Nova Scotia, New Brunswick, Quebec, Ontario, and British Columbia, accounting for approximately 16,000 adult acute care beds. The aim of the analysis was to assess VE of seasonal influenza vaccine against laboratory-confirmed influenza-related hospitalization and to characterize the burden of influenza disease in hospitalized patients during the 2011/12 influenza season. The SOS Network (now part of the Canadian Immunization Research Network; CIRN) surveillance is ongoing.

### Participants

SOS Network surveillance monitors reviewed daily admissions to medical and coronary intensive care units and medical wards. Admitted patients were eligible for the study if aged ≥16 years with community-acquired pneumonia (CAP), acute exacerbation of chronic obstructive pulmonary disease or asthma, unexplained sepsis, any other respiratory infection or diagnosis, or any respiratory or influenza-like symptoms, and screening was performed within 5 days of admission. Nasopharyngeal (NP) swabs were collected from eligible patients either as part of their clinical care or by SOS Network monitors, and tested for influenza viruses.

The study was conducted during the winter respiratory season. When the study site reported ≥two positive influenza tests or when the laboratory reported one or more positive influenza tests in two consecutive weeks, SOS monitors began screening hospital admissions 1 day per week. Patients were screened who were admitted on that day with a triage temperature ≥ 37.5 °C associated with one of the following: acute coronary syndrome, any other cardiac diagnosis, or stroke. Cardiac and stroke patients were screened in order to assess the potential burden of influenza as a precipitant. A temperature of ≥37.5 °C was used in this subgroup in an attempt to minimise false-negative influenza laboratory results associated with lag between influenza infection and related cardiac and stroke hospitalizations. This enhanced surveillance was stopped when the local laboratory reported no positive tests for influenza in two consecutive weeks. In hospitals associated with the TIBDN, influenza testing was performed 7 days per week as routine clinical practice for cardiac and stroke care.

Patients were considered an influenza case if they fulfilled the eligibility criteria and tested positive for influenza (hereafter, ‘cases’), or a test-negative control if they fulfilled the eligibility criteria and tested negative for influenza within 7 days of hospital admission (hereafter, ‘controls’). Each case was age-matched with the next ≥1 sequentially enrolled control(s) admitted to the same site 14 days before or after the admission of the case.

Patients were defined as vaccinated if they reported receipt of a 2011/12 seasonal influenza vaccine more than 14 days before the onset of their symptoms. Patients who received the 2011/2012 seasonal influenza vaccine and whose onset of illness date was unknown were initially defined as status unknown until after 14 January 2012, when they were defined as vaccinated, since the vast majority of adults in Canadian immunization programs are vaccinated before the end of the calendar year. Vaccines used in Canada contained the influenza strains recommended by the World Health Organization (WHO) for inclusion in the 2011/12 influenza season vaccines in the Northern Hemisphere: A/California/7/2009 (H1N1)-like virus; A/Perth/16/2009 (H3N2)-like virus; B/Brisbane/60/2008-like virus (Victoria lineage) [15].

### Data collection

Data were collected by SOS Network monitors via patient interview and medical record review. Standardized case report forms were used to collect detailed demographic information, medical and surgical history, details of presenting illness, hospitalization details including management, healthcare use, and ultimately discharge and 30-day post-discharge outcomes. Information about seasonal influenza vaccination status was collected by interview with the patient or their caregiver; self-reported immunization history was verified with the immunization provider or an immunization registry, provided that information was available. Study monitors contacted patients’ primary care physician/family physician and, where possible, Provincial Public Health records, to collect data on product, lot number, and date administered.

The study protocol was approved by the Research Ethics Boards of all participating institutions. All patients provided written informed consent for data and sample collection, and medical record screening in accordance with the local Research Ethics Boards requirements.

### Laboratory methods

Initial influenza testing was performed at the hospitals’ laboratory or Provincial Public Health Laboratories according to local protocols. All SOS Network sites used RT PCR, apart from one site which used viral culture. After local testing, specimens were transported to the SOS Network central laboratory at the Canadian Center for Vaccinology in Halifax, Nova Scotia where they were re-tested for influenza using RT PCR to confirm local laboratory results and for further influenza A subtype or B lineage determination.

### Statistical methods

VE was calculated as 1 minus the odds ratio (OR) of vaccination in cases compared with controls multiplied by 100. ORs were estimated by conditional logistic regression. The characteristics of cases versus controls and vaccinated versus unvaccinated cases were described and assessed using Mantel-Haenszel methods for discrete variables and linear mixed model methods for continuous variables with matched sets as random effect. No adjustment was made for multiple comparisons.

Conditional logistic regression was used to identify risk factors for influenza disease; the adjustment covariates of the VE analyses were partly selected post-hoc. VE estimates were adjusted for age and any antiviral usage prior to hospital admission. The VE estimates for the prevention of influenza-hospitalization in the final model were also adjusted using multivariate logistic regression with stepwise backward selection of covariates with *p*-values of <0.1 by univariate analysis. All matched sets with at least one case and one control without missing data for the final set of covariates were considered in the estimation of the final adjusted VE. Unadjusted and adjusted VE estimates were provided with a 95% Confidence Interval (CI).

VE against influenza-related hospitalization due to any influenza strain and also specifically due to influenza B was estimated for cases and controls admitted early in the influenza season (defined as admissions prior to the admission date of the median influenza case enrolled) and late in the influenza season (defined as admissions after the date of admission of the median influenza case enrolled).

In an exploratory analysis to assess residual bias, the final logistic regression model was used to assess VE of TIV for the prevention of respiratory viruses other than influenza. In this exploratory analysis, cases were those testing positive for a non-influenza respiratory virus by multiplex PCR and controls were those negative for both influenza and other respiratory viruses by multiplex PCR.

All analyses were performed using SAS Software version 9.2 or later (SAS Institute Inc. NC, USA).

## Results

The first patient was enrolled on 20 December 2011 and the last patient contact was on 15 July 2012. A total of 7044 patients (age ≥ 16) were screened, of whom 20.9% (*n* = 1474) were enrolled in the overall cohort, and 19.3% (*n* = 1363) were included in the VE assessment, as they had known influenza immunization status (528 cases and 835 controls). The mean age of cases and controls in the overall cohort was 67.1 years and 69.2 years, respectively. A total of 208 cases and 271 controls were aged 16–64 years (younger adult group), with a mean age of 46.3 years and 49.2 years, respectively. Demographic and clinical characteristics are shown in Table 1. Test-negative controls were more likely to have ≥1 underlying co-morbidity (*p* = 0.04), underlying cardiac disease (*p* = 0.005), and pulmonary disease (*p* = 0.021). Controls were also more likely than cases to have a body mass index (BMI) of ≥30 kg/m^2^ (*p* = 0.016) and to have been past smokers (*p* < 0.001). Influenza cases were more likely to be pregnant (*p* = 0.006). Baseline characteristics of vaccinated and unvaccinated patients are shown in Table 2. A total of 776/1363 (56.9%) patients (age ≥ 16 years) had received 2011/12 TIV.

**Table 1:** Demographic and clinical characteristics of patients aged ≥16 years (all adults group)

	Cases *N* = 528	Controls *N* = 835	*p*-value
Age, years			
Mean (SD)	67.11 (20.05)	69.17 (16.94)	0.732
Median (range)	70 (18–104)	73 (18–99)	
Age subgroups, n (%)			
16–49 years	107 (20.3)	107 (12.8)	0.118
50–64	101 (19.1)	164 (19.6)	
65–75 years	101 (31.6)	206 (36.5)	
> 75 years	219 (68.4)	358 (63.5)	
Female, n (%)	288 (54.5)	469 (56.2)	0.580
BMI, kg/m^2^			
Mean (SD)	26.51 (6.74)	26.81 (7.07)	0.407
Median (range)	25.39 (9.64–60.35)	25.73 (10.7–63.77)	
Obese (BMI ≥30 kg/m^2^), n (%)	103 (19.5)	229 (27.4)	0.016
≥1 co-morbidity, n (%)			
Cardiac disease	210 (39.8)	415 (49.7)	0.005
Vascular disease	317 (60.0)	557 (66.7)	0.093
Pulmonary disease	231 (43.8)	426 (51.0)	0.021
Smoking, n (%)			
Current	89 (16.9)	143 (17.1)	0.529
Past	143 (27.1)	334 (40.0)	<0.001
Children aged <5 years in household, n (%)			
0	456 (86.3)	763 (91.4)	0.001
1 or more	54 (10.2)	46 (5.5)	
Received 2011/12 seasonal influenza vaccine, n (%)	262 (49.6)	529 (63.4)	<0.001
Received 2010/11 seasonal influenza vaccine, n (%)	248 (47.0)	515 (61.7)	<0.001

*N* number of patients, *BMI* body mass index, *SD* standard deviation

Missing data: BMI: 40 cases (7.6%), 15 controls (1.8%); Obesity: 40 cases (7.6%), 15 controls (1.8%); Current smoking: 5 cases (0.9%), 7 controls (0.8%); Past smoking: 105 cases (19.9%), 156 controls (18.7%); Children aged <5 years in the household: 18 cases (3.4%), 26 controls (3.1%); Received 2010/11 seasonal influenza vaccine: 47 cases (8.9%), 42 controls (5.0%)

**Table 2:** Demographics and clinical characteristics of TIV-vaccinated vs unvaccinated patients ≥16 years (all adults group)

	Not vaccinated *N* = 587	TIV *N* = 776	p-value
Age, years			
Mean (SD)	61.35 (19.76)	73.68 (14.94)	<0.001
Median (range)	64 (18–98)	76 (18–104)	
Age subgroups, n (%)			
16–49 years	161 (27.4)	53 (6.8)	<0.001
50–64	143 (24.4)	122 (15.7)	
65–75 years	112 (19.1)	195 (25.1)	
> 75 years	171 (29.1)	406 (52.3)	
Female, n (%)	337 (57.4)	420 (54.1)	0.248
BMI, kg/m^2^			
Mean (SD)	26.72 (6.66)	26.68 (7.16)	0.915
Median (range)	25.49 (11.38–60.35)	25.67 (6.64–63.77)	
Obese (BMI ≥30 kg/m^2^), n (%)	136 (23.2)	196 (25.3)	0.480
≥ 1 co-morbidity, n (%)			
Cardiac disease	195 (33.2)	568 (73.2)	<0.001
Vascular disease	427 (55.0)	241 (41.1)	<0.001
Pulmonary disease	297 (50.6)	401 (51.7)	<0.001
Smoking, n (%)			
Current	127 (21.6)	105 (13.5)	<0.001
Past	162 (27.6)	315 (40.6)	<0.001
Admitted from a LTCF	15 (2.6)	70 (9.0)	<0.001
≤4 prescribed medications before admission	287 (48.9)	169 (21.8)	<0.001

*N* number of patients, *TIV* trivalent influenza vaccine, *SD* standard deviation, *BMI* body mass index, *LTCF* long-term care facility

Missing data: BMI: 29 not vaccinated (4.9%), 26 TIV-vaccinated (3.4%); Obesity: 29 not vaccinated (4.9%), 26 TIV-vaccinated (3.4%); Current smoking: 1 not vaccinated (0.2%), 11 TIV-vaccinated (1.4%); Past smoking: 131 not vaccinated (22.3%), 130 TIV-vaccinated (16.7%); Admitted from a LTCF: 0 not vaccinated (0.0%), 1 TIV-vaccinated (0.1%); ≤4 prescribed medications before admission: 10 not vaccinated (1.7%), 7 TIV-vaccinated (0.9%)

In the overall cohort, the specific 2011/12 influenza vaccine brand received could not be ascertained in 64.6% (*n* = 164) of vaccinated cases and 67.6% (*n* = 353) of vaccinated controls. Among cases/controls for whom this information was available, 30.0%/39.1% had received *Fluviral™* (GSK), 34.4%/32.5% had received *Agriflu™* (Novartis Vaccines), 28.9%/24.9% had received *Vaxigrip™* (Sanofi Pasteur), 3.3%/2.4% had received *Fluad™* (Novartis Vaccines), one vaccinated control had received Fluzone*™* (Sanofi Pasteur), and two vaccinated controls had received *FluMist™* (MedImmune). Two vaccinated cases had received another approved TIV.

### Influenza profile

**Fig. 1:**
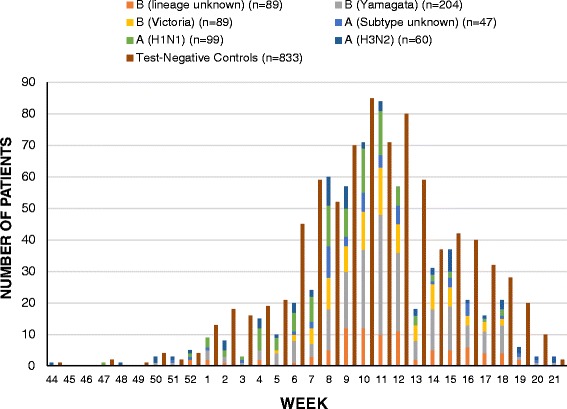
Laboratory-confirmed influenza cases and test-negative controls by week and virus subtype, November 1, 2011 to May 25, 2012

A summary of the temporal distribution of influenza-related hospitalizations admitted to SOS Network hospitals is shown in Fig. 1. Overall, in all patients ≥16 years, among the 182 cases of influenza A, 56 (30.8%) were attributable to A/H3N2, 89 (48.9%) to A/H1N1, and 37 (20.3%) were not subtyped; among the 346 influenza B cases, 188 (54.3%) were linked to B/Yamagata lineage, 81 (23.4%) to B/Victoria lineage, and 77 (22.3%) were not lineage-typed. In younger adults (16–64 years), among the 86 cases of influenza A, 13 (15.1%) were A/H3N2, and 55 (63.9%) were A/H1N1, and among the 122 cases of influenza B, 53 (43.4%) were B/Yamagata lineage and 37 (30.3%) were B/Victoria lineage.

### Vaccine effectiveness

A summary of the VE estimates for TIV in the prevention of influenza-hospitalization overall and for younger adults, respectively, are shown in Tables 3 and 4. Overall, the matched unadjusted VE for the prevention of influenza-hospitalization was 41.8% (95% CI: 26.0, 54.3), and the matched adjusted VE estimate was 42.8% (95% CI: 23.8, 57.0). In younger adults (16–64 years), the unadjusted VE for the prevention of influenza-hospitalization was 35.8% (95% CI: 4.5, 56.8) and the adjusted VE estimate was 33.2% (95% CI: −6.7, 58.2). Overall, adjusted VE against influenza A/H1N1 strains was 72.5% (95% CI: 30.5, 89.1), against influenza A/H3N2 was 86.1% (95% CI: 40.1, 96.8), against the B/Victoria lineage strain (B-lineage included in the 2011/12 TIV) was 40.5% (95% CI: −28.9, 72.6), and against the B/Yamagata lineage strain (B-lineage not included in the 2011/12 TIV) was 32.3% (95% CI: −8.3, 57.7).

**Table 3:** Vaccine effectiveness (VE) of TIV against influenza-related hospitalization in patients ≥16 years (all adults group)

	Cases	Controls	Unadjusted		Adjusted	
	N	N	VE, %	95% CI	VE, %	95% CI
All strains	528	835	41.8	26.0, 54.3	42.8^a^	23.8, 57.0
Influenza A	182	301	50.4	23.9, 67.6	55.6^b^	23.4, 74.3
A/H1N1	89	142	71.7	41.7, 86.3	72.5^c^	30.5, 89.1
A/H3N2	56	105	31.0	−45.9, 67.5	86.1^d^	40.1, 96.8
Influenza B	346	534	37.3	16.0, 53.2	36.2^e^	10.0, 54.7
B/Victoria	81	126	47.1	3.4, 71.0	40.5^f^	−28.9, 72.6
B/Yamagata	188	292	28.7	−5.4, 51.8	32.3^g^	−8.3, 57.7

*N* number of patients, *CI* Confidence Interval, *TIV* trivalent influenza vaccine, *VE* vaccine effectiveness

Covariate (p-value in model):

^a^ Influenza vaccination (<0.001), age (0.378), anti-viral use before admission (0.902), admission from long term care facility (0.005), obesity (0.119), exposed to children aged <5 years in household (0.034), current or past smoker (0.000), medications before onset of illness (0.011);

^b^ Influenza vaccination (0.004), age (0.264), anti-viral use before admission (0.766), admission from long term care facility (0.592), obesity (0.633), exposed to children aged <5 years in household (0.157), current or past smoker (0.376), medications before onset of illness (0.191);

^c^ Influenza vaccination (0.006), age (0.093), anti-viral use before admission (0.814), admission from long term care facility (0.993), obesity (0.199), exposed to children aged <5 years in household (0.311), current or past smoker (0.188), medications before onset of illness (0.046);

^d^ Influenza vaccination (0.008), age (0.022), admission from long term care facility (0.419), obesity (0.998), exposed to children aged <5 years in household (0.046), current or past smoker (0.571), medications before onset of illness (0.560);

^e^ Influenza vaccination (0.010), age (0.791), anti-viral use before admission (0.471), admission from long term care facility (0.001), obesity (0.143), exposed to children aged <5 years in household (0.096), current or past smoker (0.000), medications before onset of illness (0.023);

^f^ Influenza vaccination (0.188), age (0.215), admission from long term care facility (0.794), obesity (0.750), exposed to children aged <5 years in household (0.184), current or past smoker (0.025), medications before onset of illness (0.093);

^g^ Influenza vaccination (0.103), age (0.021), anti-viral use before admission (0.456), admission from long term care facility (0.002), obesity (0.319), exposed to children aged <5 years in household (0.005), current or past smoker (0.004), medications before onset of illness (0.087);

**Table 4:** Vaccine effectiveness (VE) of TIV against influenza-related hospitalization in patients ≥16–64 years (younger adults group)

	Cases	Controls	Unadjusted		Adjusted	
	N	N	VE, %	95% CI	VE, %	95% CI
All strains	208	271	35.8	4.5, 56.8	33.2^a^	−6.7, 58.2
*Influenza A*	86	110	48.5	1.4, 73.1	42.2^b^	−24.2, 73.1
A/H1N1	55	68	57.8	2.7, 81.7	68.7^c^	6.4, 89.5
A/H3N2	13	18	38.2	−599.5, 94.5	–	–
*Influenza B*	122	161	26.2	−22.2, 55.4	25.4^d^	−35.4, 58.9
B/Victoria	37	48	25.6	−82.8, 69.7	9.7^e^	−161.6, 68.9
B/Yamagata	53	70	16.6	−81.1, 61.6	36.9^f^	−72.5, 76.9

*N* number of patients, *CI* Confidence Interval, *TIV* trivalent influenza vaccine, *VE* vaccine effectiveness

Covariate (p-value in model):

^a^ Influenza vaccination (0.092), age (0.018), anti-viral use before admission (0.881), seniors including patient living in dwelling (0.020), pregnancy (0.045);

^b^ Influenza vaccination (0.160), age (0.192), anti-viral use before admission (0.874) seniors including patient living in dwelling (0.188), pregnancy (0.991);

^c^ Influenza vaccination (0.038), age (0.173), anti-viral use before admission (0.895) seniors including patient living in dwelling (0.201), pregnancy (0.994);

^d^ Influenza vaccination (0.335), age (0.045), seniors including patient living in dwelling (0.046), pregnancy (0.099);

^e^ Influenza vaccination (0.850), age (0.054), seniors including patient living in dwelling (0.888), pregnancy (0.996);

^f^ Influenza vaccination (0.370), age (0.783), seniors including patient living in dwelling (0.037), pregnancy (0.740)

VE of TIV against influenza-related hospitalization during early-season and during late-season are shown in Table 5. VE against influenza-related hospitalization during early-season was 54.4% (95% CI: 29.7, 70.4), and during late-season was 29.7% (95% CI: −5.3, 53.1); VE against influenza-related hospitalization due to influenza B during early-season was 44.8% (95% CI: 0.3, 69.5), and during late-season was 33.1% (95% CI: −5.2, 57.5). Among younger adults (16–64 years), VE against influenza B years during early-season was 67.1% (95% CI: 2.7, 88.9), and during late-season was −52.3% (95% CI: −251.6, 34.0).

**Table 5:** Vaccine effectiveness (VE) of TIV against influenza-related hospitalization by early/late season in patients ≥16 years, ≥16–64 years, and ≥65 years

	Influenza Type	Cases	Controls	Unadjusted		Adjusted	
		N	N	VE, %	95% CI	VE, %	95% CI
Age ≥ 16 years (all ages)	Early season						
	All strains	265	423	48.6	27.9, 63.3	54.4^a^	29.7, 70.4
	Influenza B	148	230	41.0	8.4, 62.1	44.8^b^	0.3, 69.5
	Late season						
	All strains	263	412	33.8	6.6, 53.0	29.7^c^	−5.3, 53.1
	Influenza B	198	304	34.1	2.8, 55.4	33.1^d^	−5.2, 57.5
Age ≥ 16–64 years (younger adults)	Early season						
	All strains	109	131	59.0	27.6, 76.8	65.7^e^	28.2, 83.6
	Influenza B	55	68	60.1	12.7, 81.8	67.1^f^	2.7, 88.9
	Late season						
	All strains	99	140	−8.5	−95.0, 39.6	−36.1^g^	−171.2, 31.7
	Influenza B	67	93	−27.7	−175.5, 36.7	−52.3^h^	−251.6, 34.0
Age ≥ 65 years (older adults)	Early season						
	All strains	156	292	41.2	9.8, 61.7	52.5^i^	8.0, 75.5
	Influenza B	93	162	27.4	−25.9, 58.2	36.3^j^	−45.1, 72.0
	Late season						
	All strains	164	272	48.6	21.3, 66.4	62.4^k^	29.9, 79.9
	Influenza B	131	211	50.9	21.2, 69.4	69.3^l^	37.4, 84.9

*N* number of patients, *CI* Confidence Interval, *TIV* trivalent influenza vaccine

Early season defined as admissions prior to the admission date of the median influenza case enrolled; Late season defined as admissions after the date of admission of the median influenza case enrolled

Covariate (*p* value in model):

^a^ Influenza vaccination (<0.001), age (0.116), antiviral use before onset of illness (0.821), admission from long term care facility (0.001), obesity (0.017), exposed to children aged <5 years in household (0.536), current or past smoker (0.015), medications before onset of illness (0.325);

^b^ Influenza vaccination (0.049), age (0.099), antiviral use before onset of illness (0.992), admission from long term care facility (<0.001), obesity (0.020), exposed to children aged <5 years in household (0.447), current or past smoker (0.001), medications before onset of illness (0.515);

^c^ Influenza vaccination (0.088), age (0.383), antiviral use before onset of illness (0.988), admission from long term care facility (0.960), obesity (0.859), exposed to children aged <5 years in household (0.030), current or past smoker (0.003), medications before onset of illness (0.007);

^d^ Influenza vaccination (0.082), age (0.150), antiviral use before onset of illness (0.992), admission from long term care facility (0.884), obesity (0.764), exposed to children aged <5 years in household (0.060), current or past smoker (0.002), medications before onset of illness (0.018);

^e^ Influenza vaccination (0.005), age (0.035), antiviral use before onset of illness (0.998), seniors including patient living in the dwelling (0.129), pregnancy (0.994);

^f^ Influenza vaccination (0.045), age (0.021), seniors including patient living in the dwelling (0.061), pregnancy (0.995), (no patients used antivirals before onset of illness);

^g^ Influenza vaccination (0.381), age (0.383), antiviral use before onset of illness (0.992), seniors including patient living in the dwelling (0.046), pregnancy (0.264);

^h^ Influenza vaccination (0.324), age (0.993), seniors including patient living in the dwelling (0.173), pregnancy (0.188), (no patients used antivirals before onset of illness);

^i^ Influenza vaccination (0.027), age (0.151), antiviral use before onset of illness (0.991), frailty index prior to admission (0.057), exposed to children aged <5 years in household (0.179), medications before onset of illness (0.051);

^j^ Influenza vaccination (0.283), age (0.220), antiviral use before onset of illness (0.995), frailty index prior to admission (0.030), exposed to children aged <5 years in household (0.399), medications before onset of illness (0.031);

^k^ Influenza vaccination (0.002), age (0.992), antiviral use before onset of illness (0.993), frailty index prior to admission (0.076), exposed to children aged <5 years in household (0.107), medications before onset of illness (0.397);

^l^ Influenza vaccination (0.001), age (0.673), antiviral use before onset of illness (0.993), frailty index prior to admission (0.043), exposed to children aged <5 years in household (0.467), medications before onset of illness (0.339)

The unadjusted VE for TIV against hospitalization in patients with non-influenza respiratory viruses (*n* = 140 cases) compared with patients who were negative for influenza and other viruses (*n* = 469 controls) was −5.0% (95% CI: -54.9, 28.8), and the adjusted VE was −19.9% (95% CI: -83.6, 21.6).

## Discussion

In this study, the adjusted VE estimate of TIV against influenza-hospitalization in all adults aged ≥16 years was moderate (42.8%; 95% CI: 23.8, 57.0), although VE tended to be lower in younger adults aged 16–64 years (33.2%; 95% CI: −6.7, 58.2). More than three-quarters of the influenza-hospitalizations occurred later in the season during February, March and April, and VE of TIV for preventing influenza-related hospitalizations was lower in late-season relative to early-season (VE: 29.7%, 95% CI: −5.3, 53.1 and VE: 54.4%, 95% CI: 29.7, 70.4, respectively).

The 2011/2012 season in Canada represented an unusual opportunity to assess VE because in addition to the two influenza A strains (H1N1 and H3N2), influenza B strains from both the Yamagata-lineage and Victoria lineages were also co-circulating. The season was relatively mild, peaked late between February and April, and was characterized by a fairly balanced circulation of both influenza A and B viruses [13]. About 53.4% of viruses detected by national surveillance in Canada during the 2011/12 season were influenza B, and about half of the B viruses tested by Canada’s National Microbiology Laboratory (NML) were antigenically similar to the vaccine strain (B/Brisbane/60/2008-like virus), while the other half were similar to the B lineage not included in the TIV (B/Wisconsin/01/2010-like virus) [13]. Among influenza A viruses tested by the NML, more than 90% of influenza A viruses were antigenically similar to the vaccine strains (A/California/07/2009 H1N1-like virus and A/Perth/16/2009 H3N2-like virus) [13].

Compared to national surveillance in Canada, in the SOS Network during the 2011/2012 season the virus predominantly leading to hospitalisation was influenza B, with the co-circulation of B/Victoria (81 cases) and B/Yamagata (188 cases) lineage viruses, as well as both influenza A viruses; A/H1N1 (89 cases) and A/H3N2 (56 cases). Although the adjusted VE of TIV against influenza B-related hospitalization was statistically significant in the all adults cohort (≥16 years) (VE: 36.2%; 95% CI: 10.0, 54.7), the VE against influenza B-related hospitalization in younger adults (16–64 years) was not (VE: 25.4%; 95% CI: −35.4, 58.9). The adjusted VE estimates in all adults cohort (≥16 years) against B/Victoria (vaccine-matched) and B/Yamagata (not included in TIV) related hospitalizations were 40.5% and 32.3%, respectively, and in younger adults were 9.7 and 36.9%, respectively, but none of these B lineage VE estimates were statistically significant. While the B/Yamagata component was not included in the TIV, VE estimates for preventing B/Yamagata-related hospitalizations were similar to VE estimates for preventing B/Victoria-related hospitalizations, suggesting a possibility of cross-protection of TIV between influenza B lineages, however this was not statistically significant. It is possible that individuals could have been exposed to the B/Yamagata lineage during natural infection in the preceding seasons. Adjusted VE estimates were statistically significant for protection against hospitalization with A/H1N1 in the all adults cohort (72.5%) and in younger adults (68.7%), and against A/H3N2 in the all adults cohort (86.1%). There were too few cases of A/H3N2 in younger adults (13 cases) to estimate VE.

Other sentinel surveillance studies conducted during the 2011/12 season in North America and Europe also reported the co-circulation of influenza A viruses and both influenza B lineage viruses. [16–18]. In a test-negative case-control study conducted in Canada in the 2011/12 season, the adjusted VE against medically-attended influenza for TIV versus unvaccinated subjects against any influenza strain was 59%, for A/H1N1 was 80%, and for vaccine-matched B/Victoria was 71%; however, protection was suboptimal for the circulating A/H3N2 variants and the B/Yamagata strain (not included in TIV), with adjusted VE estimates of 51 and 27%, respectively [18]. Sub-optimal protection against A/H3N2-hospitalization during the 2011/12 season was also observed in Europe, where the circulating A/H3N2 virus was reported to have drifted genetically and antigenically away from the vaccine strain [19].

Prospective studies of laboratory-confirmed influenza infections have suggested that influenza vaccination may provide higher protection against more severe influenza outcomes such as hospitalization and intensive care unit (ICU) admission. During the 2006/2007–2008/2009 influenza seasons, in community-dwelling people aged ≥50 years in the US, the adjusted VE of influenza vaccination against influenza-hospitalization was 61.2% [20]. Furthermore, in a European study in 2010/11, among the general population, the adjusted VE estimate of TIV against medically-attended influenza was 75%, against influenza-hospitalization was 60%, while against severe influenza cases resulting in ICU admission or death, the VE was 89% [21]. In our study, although VE of TIV was significant for the prevention of hospitalization in the overall adult cohort, it was not statistically significant in younger adults. The increased VE observed in the elderly may have been associated with better vaccine protection against severe cases of influenza in older adults compared with less-severe influenza complications in younger adults. Additionally, the proportion of immunocompromised patients in the younger cohort was particularly high in the SOS Network this season (22.6% were considered immunocompromised), and given that studies have demonstrated decreased immune responses to influenza vaccination in immunocompromised patients, this could potentially be contributing to the decreased VE observed in this group [22]. This analysis was not powered to compare VE against intensive care unit (ICU) admission, mechanical ventilation or death, and the VE estimate of TIV was not significant in either cohort for the prevention of death or ICU admission/mechanical ventilation (data not shown).

Differences in TIV early-season VE (VE: 54.4%, 95% CI: 29.7, 70.4) versus late-season VE (VE: 29.7%, 95% CI: −5.3, 53.1) are likely attributed to the delayed influenza B peak that Canada often sees during its influenza seasons. Generally, in Canada, influenza A peaks earlier in the season, followed by a later influenza B peak. Given influenza B Yamagata circulated more heavily in the late-season and it was not included in the TIV, this discrepancy in VE by time in the season is understandable. Although influenza A has often been hypothesized to contribute to more serious influenza infections, a recent report over eight influenza seasons in the US demonstrated no significant differences in proportions of serious outcomes in patients hospitalized with influenza A vs B, despite influenza B contributing far fewer cases than influenza A [23]. Among younger adults, VE against influenza B years during late-season was not significantly protective at −52.3% (95% CI: −251.6, 34.0), although a lack of power contributed to the wide CIs observed. However, approximately 22.6% of adults 16–64 years were immunocompromised (had cancer, haematopoetic or solid organ transplant, HIV, or were taking immunosuppressive medications). About 12.1% of these patients had cancer, and 13.6% were on immunosuppressive medications (data not shown), which could be contributing to decreased VE in this age bracket. Conversely, late-season VE against all-strain influenza and influenza B was highly effective in older adults ≥65 years (VE: 62.4%, VE: 69.3%, respectively). One explanation for this could be that the older adult age group had residual immunity to the B Yamagata lineage that was not included in the TIV and circulated late-season, while younger adults may have lacked this previously acquired immunity to B Yamagata and had no protection conferred by vaccine, resulting in the poor and non-effective VE observed.

One of the strengths of this study was the assessment of influenza VE for the prevention of non-influenza respiratory viruses. Given the observational nature of the study, there is potential for unmeasured confounders to influence VE estimates. We were unable to demonstrate any impact of TIV on hospitalization for non-influenza respiratory viruses (VE: −19.9%, 95% CI: -83.6, 21.6). This suggests that there is little important residual bias in the study design, as one would not expect to observe protection of TIV against other laboratory-confirmed non-influenza respiratory viruses. Other strengths to our study include the sentinel approach, which provides consistency within a large-scale observational setting, and in Canada has been used to develop a rigorous test-negative case-control design over multiple seasons [24–28]. Further strengths were the use of multiplex RT PCR methods to characterize influenza viruses as well as other respiratory co-infections, including the determination of influenza B lineages, and the collection of detailed information on confounding factors with subsequent adjustment of VE estimates.

The main limitations of the study were the observational design and the relative infrequency of hospitalization for influenza among younger healthy adults, which limit the ability to generate sufficiently powered data. Further factors which limit the generalizability of the results across seasons include the inability to entirely predict viral circulation and subsequently vaccine strain match or mismatch within a given season in light of differences in viral epidemiology across regions.

## Conclusions

This study evaluated the VE of TIV for preventing influenza-related hospitalizations in Canada using a large, prospective, sentinel surveillance network and a test-negative case-control study design. These results suggest that TIV was highly effective against A viruses and moderately effective against B viruses during a mild season in Canada which was characterised by co-circulation of four influenza strains, including both Yamagata and Victoria B-lineages. These findings underscore the need to provide VE assessment by subtype/lineage as well as the timing of vaccination (early season vs late season) to accurately evaluate vaccine performance and thus guide public health decision-making. The SOS Network surveillance provides a unique opportunity for ongoing evaluation of seasonal influenza VE for preventing hospitalizations and other severe outcomes in Canada.

## Abbreviations

CAP: Community-acquired pneumonia
ICU: Intensive care unit
NML: National Microbiology Laboratory
NP: Nasopharyngeal
PCIRN: Public Health Agency of Canada/Canadian Institutes of Health Research Influenza Research Network
PCR: Polymerase chain reaction
RT PCR: Reverse transcription polymerase chain reaction
SOS: Serious Outcomes Surveillance
TIBDN: Toronto Invasive Bacterial Diseases Network
TIV: Trivalent influenza vaccine
VE: Vaccine effectiveness
WHO: World Health Organization
